# Efficacy of a Russian-backbone live attenuated influenza vaccine among young children in Bangladesh: a randomised, double-blind, placebo-controlled trial

**DOI:** 10.1016/S2214-109X(16)30200-5

**Published:** 2016-10-13

**Authors:** W Abdullah Brooks, K Zaman, Kristen D C Lewis, Justin R Ortiz, Doli Goswami, Jodi Feser, Amina Tahia Sharmeen, Kamrun Nahar, Mustafizur Rahman, Mohammed Ziaur Rahman, Burc Barin, Muhammad Yunus, Alicia M Fry, Joseph Bresee, Tasnim Azim, Kathleen M Neuzil

**Affiliations:** a International Centre for Diarrhoeal Disease Research, Bangladesh, Dhaka, Bangladesh; b International Health, Johns Hopkins University, Baltimore, MD, USA; c PATH, Seattle, WA, USA; d Departments of Global Health and Medicine, University of Washington, Seattle, WA, USA; e The EMMES Corporation, Rockville, MD, USA; f Influenza Division, National Center for Immunization and Respiratory Diseases, Centers for Disease Control and Prevention, Atlanta, GA, USA

## Abstract

**Background:**

The rates of influenza illness and associated complications are high among children in Bangladesh. We assessed the clinical efficacy and safety of a Russian-backbone live attenuated influenza vaccine (LAIV) at two field sites in Bangladesh.

**Methods:**

Between Feb 27 and April 9, 2013, children aged 2–4 years in urban Kamalapur and rural Matlab, Bangladesh, were randomly assigned in a 2:1 ratio, according to a computer-generated schedule, to receive one intranasal dose of LAIV or placebo. After vaccination, we monitored children in weekly home visits until Dec 31, 2013, with study clinic surveillance for influenza illness. The primary outcome was symptomatic, laboratory-confirmed influenza illness due to vaccine-matched strains. Analysis was per protocol. The trial is registered with ClinicalTrials.gov, number NCT01797029.

**Findings:**

Of 1761 children enrolled, 1174 received LAIV and 587 received placebo. Laboratory-confirmed influenza illness due to vaccine-matched strains was seen in 93 (15·8%) children in the placebo group and 79 (6·7%) in the LAIV group. Vaccine efficacy of LAIV for vaccine-matched strains was 57·5% (95% CI 43·6–68·0). The vaccine was well tolerated, and adverse events were balanced between the groups. The most frequent adverse events were tachypnoea (n=86 in the LAIV group and n=54 in the placebo group), cough (n=73 and n=43), and runny nose (n=68 and n=39), most of which were mild.

**Interpretation:**

This single-dose Russian-backbone LAIV was safe and efficacious at preventing symptomatic laboratory-confirmed influenza illness due to vaccine-matched strains. LAIV programmes might reduce the burden of influenza illness in Bangladesh.

**Funding:**

The Bill & Melinda Gates Foundation.

## Introduction

The seasonal patterns, incidence, and severity of influenza virus infection are poorly defined in many tropical regions.^1^ In Bangladesh, over a decade of surveillance data show that influenza virus circulates during most months of the year and that infection is a frequent cause of febrile illness and lower respiratory tract infections in young children.^2^, ^3^, ^4^, ^5^, ^6^, ^7^ Furthermore, in urban Kamalapur, which has a background clinical pneumonia incidence of 500 cases per 1000 child-years, community-based surveillance identified that 10% of children younger than 5 years with clinical pneumonia are positive for influenza virus.^4^ Thus, prevention of influenza illness could have a substantial effect on childhood morbidity.

Live attenuated influenza vaccines (LAIVs) are attractive for use in young children because they may be delivered intranasally and have good efficacy. In a WHO-sponsored technology transfer programme, several manufacturers are developing reassortant LAIVs based on A/Leningrad/17 and B/USSR/60 master donor viruses. This initiative could provide affordable supplies of influenza vaccine in Bangladesh and other low-resource countries. In 2012, we did a phase 2 study involving 300 children aged 24–59 months in urban Bangladesh that supported the safety of a Russian-backbone LAIV.^8^ We have now done a clinical efficacy trial to investigate the benefit of a single-dose Russian-backbone LAIV in children in urban and rural sites in Bangladesh.

## Methods

### Study design

This was a two-site, randomised, double-blind, placebo-controlled, parallel-group clinical trial. The study was done at two demographic surveillance sites of the International Centre for Diarrhoeal Disease Research, Dhaka, Bangladesh: one in urban Kamalapur and one in rural Matlab. The study was approved by the ethics review committees of the International Centre for Diarrhoeal Disease Research, and by the Western Institutional Review Board, Puyallup, WA, USA. Participant safety was overseen by an independent international data safety monitoring board that was convened by the study's sponsor, PATH, Seattle, WA, USA, and a local data safety monitoring board convened by the International Centre for Diarrhoeal Disease Research. The study complied with the principles of the Declaration of Helsinki, and was done in compliance with Good Clinical Practice guidelines.

**Research in context**

**Evidence before this study**

Cold-adapted, temperature-sensitive live attenuated influenza vaccines (LAIVs) have been developed in the USA and Russia. These vaccines contain viruses produced by reassorting master donor viruses (A/Ann Arbor/6/60 and B/Ann Arbor/1/66 or A/Leningrad/134/17/57 and B/USSR/69/60, respectively) with viruses recommended by WHO or the US Public Health Service. Decisions are based on the expected circulation in the forthcoming northern or southern hemisphere influenza season. LAIVs based on Ann-Arbor-derived strains are approved for use in children ages 2 years and older, and those based on the Russian-derived strains are approved for single-dose administration to children aged 3 years and older. Through a WHO agreement, manufacturers in several developing countries have access to Russian-derived vaccine strains for production of their own LAIVs. The Serum Institute of India, Pune, India, is one such manufacturer. Its LAIV is approved for use in children aged 2 years and upwards. Policy decisions and sustainable use of LAIVs in developing countries will depend on the generation of data that show their efficacy in representative populations. We searched PubMed from Jan 1, 1980, to Jan 1, 2016, for efficacy trials assessing protection achieved with the use of LAIVs in children, using the search terms, “human influenza”, “vaccines, attenuated”, and “children”. LAIVs have been studied primarily in developed countries in Europe and Asia and in the USA. Use of the Ann-Arbor-derived LAIV in children younger than 72 months was studied, with culture confirmation of illness, in several trials from 1996 to 2005 in these regions plus one study in South Africa in children aged from 6 up to 36 months. These studies were done under Good Clinical Practice guidelines. In nearly all studies, protection was significant. Before those studies, five studies of Russian-derived LAIVs had been done in the Soviet Union and Cuba, primarily in 1986–91. These included nearly 28 000 children aged 3–6 years. These studies used serological confirmation of illness and were done before Good Clinical Practice standards were introduced for trials. Moreover, except for one study in Senegal, no efficacy trials of Russian-derived LAIVs had been done in developing countries. We found one case-control study assessing the clinical protection of a single-dose monovalent Serum Institute of India LAIV containing vaccine virus antigenically similar to pandemic A/H1N1 (2009).

**Added value of this study**

Lower respiratory tract disease caused by viral infections is common among young children in Bangladesh. LAIVs could potentially reduce some of this morbidity by preventing primary influenza disease, its complications, or both. The feasibility of influenza immunisation in Bangladesh would be increased if a single-dose LAIV provided protection. In our randomised trial of LAIV done exclusively among children in Bangladesh, we showed significant protection against PCR-confirmed influenza illness in children aged 2–4 years.

**Implications of all the available evidence**

Russian-backbone LAIV provided laboratory-confirmed protection in a low-resource setting, in rural and urban sites. This finding suggests that single-dose LAIVs could be the optimum choice for protection against influenza virus in paediatric populations in Asia. We are uncertain why the single-dose LAIV was protective in Bangladesh but not in Senegal, where no efficacy was seen, but this difference emphasises the variability in responses to influenza vaccine and the need for testing in multiple populations and, ideally, in multiple seasons.

### Participants

Healthy children aged 2–4 years who lived in either of the two demographic surveillance areas were eligible for enrolment if a parent or legal guardian provided written informed consent and the family was not expecting to migrate out of the area during the study. In Kamalapur, we approached parents of eligible children during weekly field visits in households previously selected by cluster randomisation for active surveillance. In Matlab, we approached parents of eligible children as determined by our demographic surveillance system by visiting their households. Exclusion criteria were the same as in the previous phase 2 study,^8^ and included serious, active medical disorders and having previously received any influenza vaccine. The complete list of eligibility criteria is provided in the appendix.

### Randomisation and masking

The random allocation sequence was computer generated by PATH staff not involved with the trial, using a ratio for LAIV and placebo of 2:1 and block sizes of three. The sequence was delivered to the Serum Institute of India, Pune, India, where it was used to label the vaccine and placebo syringes, which were identical in appearance except for the allocation numbers. The labelled syringes of vaccine and placebo were shipped to Bangladesh for use.

### Study vaccine and placebo

The syringes were used to fill spray devices with vaccine or placebo. Each dose was 0·5 mL, with half delivered into each nostril. The LAIV was 2012–13 Northern Hemisphere formulation (Nasovac-S, Serum Institute of India, lot 167E2002) and contained A/California/7/2009 (H1N1)-like, A/Victoria/361/2011 (H3N2)-like, and B/Wisconsin/1/2010 (Yamagata lineage)-like reassortants. The placebo was the vaccine vehicle without the virus components (Serum Institute of India, lot E9001PCB). The sprays were administered to each child according to the manufacturer's recommendations.^9^

### Procedures

Participants were given one dose of study vaccine or placebo in the clinic and asked to remain for 30 min after administration, under the supervision of trained study nurses. Field workers did daily home visits up to day 4 after vaccination to monitor solicited events, unsolicited events, protocol-defined wheezing illness (PDWI), and serious adverse events (SAEs). If children presented at the clinic on days 4–7 with symptoms, these were included in solicited events. Thereafter children were monitored weekly at home by trained field workers. All children identified through home-visit surveillance as meeting protocol-defined criteria for physician assessment (signs of illness) were assessed by a study physician in the clinic according to standardised criteria. If children met protocol-defined criteria for specimen collection and presented within 7 days of illness onset, a nasopharyngeal wash specimen was collected for testing by real-time RT-PCR for evidence of influenza virus infection, according to the WHO laboratory protocol.^10^

The physician assessment criteria included the presence of at least one major or two minor signs. Major signs were fever (axillary temperature ≥38·0°C), tachypnoea (≥40 breaths per min), danger signs (chest wall indrawing, lethargy, cyanosis, inability to drink, convulsions), difficulty breathing, noisy breathing, ear pain, or ear discharge. Minor signs were subjective fever (feverishness), cough, rhinorrhoea, sore throat, myalgia or arthralgia, chills, headache, irritability or decreased activity, or vomiting. The criteria for specimen collection were two or more of axillary temperature 37·5°C or higher, cough, sore throat, and runny nose or nasal congestion present on the same day, or any of fever (axillary temperature ≥38·0°C), upper respiratory illness (axillary temperature ≥37·5°C with cough and rhinorrhoea), pneumonia, acute otitis media, meningitis, or sepsis, as diagnosed by the physician.^4^, ^11^

Assays were done at the International Centre for Diarrhoeal Disease Research, Bangladesh, as previously described.^12^, ^13^ Antigenic characterisation of positive samples was done at the virology laboratory at the International Centre for Diarrhoeal Disease Research.^13^ Genotyping was done to distinguish vaccine virus from wild-type virus at the Department of Virology, Institute of Experimental Medicine, Saint Petersburg, Russia, using specimens that were PCR positive within 14 days of vaccination.^14^

### Outcomes

The primary efficacy endpoint was symptomatic, laboratory-confirmed influenza virus infection with vaccine-matched strains up to December, 2013. The secondary efficacy endpoint was symptomatic, laboratory-confirmed influenza virus infection with any influenza virus strain.

Safety endpoints included immediate reactions occurring within 30 min of taking the vaccine, solicited reactions (nasal congestion, runny nose, ear pain, cough, sore throat, headache, fever, tachypnoea, muscle or joint pain, chills, irritability or decreased activity, and vomiting) and unsolicited adverse events occurring in the first 4 days after vaccination, as assessed by daily home visits, and up to day 7, as captured in the clinic. Severity of adverse events was graded as mild, moderate, severe, and life threatening. PDWI was defined as an illness meeting the physician assessment criteria and characterised by a long, high-pitched whistling or musical sound on expiration heard by auscultation over the lung fields. Severity of PDWI was graded by study physicians as mild (wheezing illness without other findings associated with moderate or greater severity disease), moderate (nasal flaring, chest in-drawing, or pulse oximetry ≥90% to <95%), severe (dyspnoea at rest causing inability to perform usual activities or pulse oximetry <90%), or life threatening. This definition was designed to be similar to the definitions of medically important wheezing used in previous trials^8^, ^15^ and to exclude incidental wheezing illness without other clinically important signs or symptoms.

### Statistical analyses

Assuming 60% efficacy, we calculated that 57 symptomatic, laboratory-confirmed influenza virus infections would be needed to test the hypothesis that LAIV efficacy was greater than 0 with a one-sided type I error of less than 2·5% and power of at least 90% (exact type I error and power 1·9% and 91·5%, respectively). On the basis of this number and assuming 6% influenza illness incidence in the placebo group and that 90% of children would be assessable, we estimated that we would need a total sample size of 1761 enrolled and vaccinated children.

The primary objective was to estimate the efficacy of LAIV to reduce the incidence of symptomatic, laboratory-confirmed influenza virus infection with vaccine-matched strains, compared with placebo. Vaccine efficacy, expressed as a percentage, was defined as 1 minus the relative rate of influenza in the LAIV group compared with that in the placebo group. Efficacy with 95% CIs was computed with a binomial distribution of LAIV cases. We used Fisher's exact test to obtain two-sided p values for the test of the null hypothesis of zero vaccine efficacy. Primary efficacy analyses and summaries were done on a per-protocol basis. The per-protocol analysis set included all children who met the inclusion criteria, were randomised, and received one dose of study vaccine or placebo, and who remained in the study area for at least 8 days after vaccination. Analyses were based on the first case of influenza occurring from study day 8 onwards until Dec 31, 2013. Supportive analyses were done in the total vaccinated cohort (ie, all children who were randomised and received one dose of vaccine or placebo, irrespective of how long they stayed in the study area). All analyses excluded samples identified as containing vaccine virus.

Safety was assessed in the total vaccinated cohort and was described as the proportion (95% CI) of children who had any reaction or adverse event. Differences between groups were calculated, including two-sided p values, with Fisher's exact test. Statistical analyses were done with SAS version 9.3. This study is registered with ClinicalTrials.gov, number NCT01797029.

### Role of the funding source

The funder had no role in the study, data collection, data analysis, data interpretation, or the writing of the report. The corresponding author had full access to all the data in the study and had final responsibility for the decision to submit for publication.

## Results

### Study population

**Figure 1:**
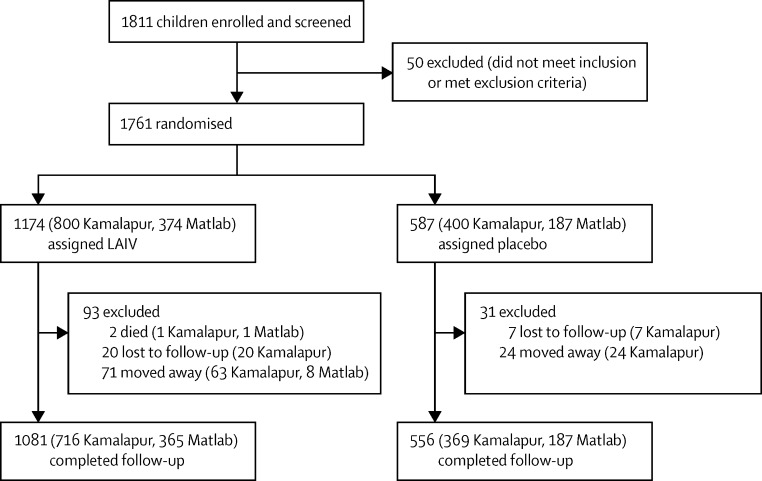
Trial profile LAIV=live attenuated influenza vaccine.

Of 1811 children enrolled, 1761 received vaccine or placebo between Feb 27 and April 9, 2013 (total vaccinated analysis set), of whom 1637 were followed up for study outcomes until Dec 31, 2013 (figure 1). No participants were lost to follow-up before day 8. 1637 (92·9%) of study participants completed home follow-up visits to December, 2013, 1081 (92·0%) of those who received LAIV and 556 (94·7%) of those who received placebo (p=0·048).

Baseline demographic characteristics and medical history were similar in the two study groups (table 1), although differences were noted between the urban Kamalapur site and the rural Matlab site. Nearly twice as many children had severe stunting in Kamalapur as in Matlab, and previous medical treatment for asthma or wheezing illness was 31·3% in Kamalapur, including 6·5% who were admitted to hospital, compared with no children in Matlab.

**Figure 2:**
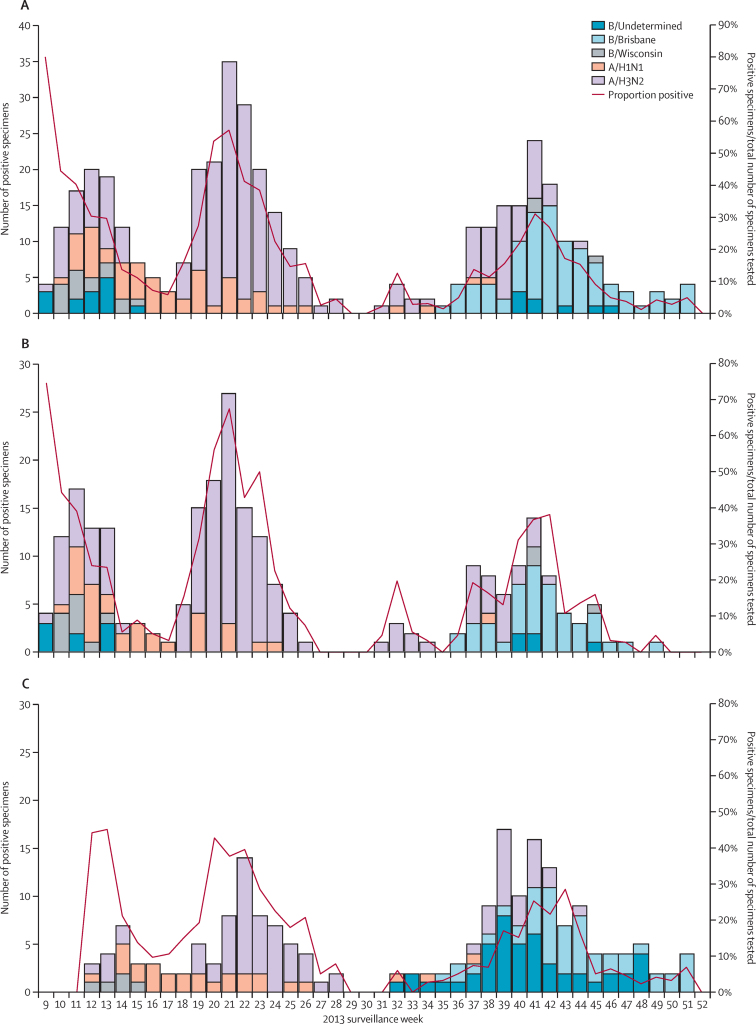
Influenza circulation in the study area overall and in Kamalapur and Matlab, by type and subtype or lineage, in weeks 9–52 of 2013 (A) The whole study area. (B) Kamalapur. (C) Matlab. Circulating and vaccine B strains were mismatched and, therefore, did not contribute to vaccine efficacy

Influenza circulated in the study area from February to November, 2013 (figure 2). Symptomatic, laboratory-confirmed influenza illness due to vaccine-matched influenza viruses from 8 days after vaccination onwards was seen in 79 (6·7%) participants in the LAIV group and 93 (15·8%) participants in the placebo group, giving a vaccine efficacy of 57·5% (95% CI 43·6–68·0; table 2). In the strain-specific analysis in the per-protocol population (table 2), efficacy was 50–60% for the H3N2 and H1N1 strains. Only three children had vaccine-matched B/Yamagata strains isolated during the study. The attack rate for the mismatched B/Victoria lineage was statistically similar in the two study groups and, therefore, there was no vaccine efficacy against mismatched B lineage viruses. When all strains of influenza virus were analysed in the per-protocol population, including the mismatched B strains, vaccine efficacy was 41·0% (table 2). Results were similar in the total vaccinated cohort (appendix). When vaccine efficacy was assessed by study site, the rural Matlab site had higher influenza illness attack rates and higher vaccine efficacy than the urban Kamalapur site (table 2). In a post-hoc analysis, children with a history of asthma or wheezing illness in Kamalapur had a lower point estimate of efficacy than children without such a history (table 2).

No immediate reactions were seen after vaccine or placebo were administered. In the 7 days after receiving the study drug or placebo, the most common local and systemic events were runny nose, cough, and tachypnoea (table 3). Nearly all reactions were mild and similar proportions of children were affected in the vaccine and placebo groups. Among children with any history of asthma or wheezing illness, the frequency of PDWI was very similar in the LAIV and placebo groups, and among those without such a history, more in the placebo group than in the LAIV group had PDWI (table 3). No proportions of children with PDWI were significantly higher in the LAIV than in the placebo group when assessments were done by age group, site, site and age group, or history of asthma or wheezing illness at baseline. Two children died in the vaccine group during the study, both owing to drowning. Neither death was deemed to be related to vaccination.

## Discussion

This prospective trial of a single-dose Russian-backbone LAIV with intranasal delivery showed vaccine efficacy in children in Bangladesh with a good safety profile. These findings add important information to the data available on this vaccine because the original licensing studies done in Russia took place before PCR was used to confirm influenza virus infection, and licensing in India did not require laboratory-confirmed prospective efficacy studies. LAIV was efficacious against infection with circulating influenza H1N1pdm09 and H3N2 viruses, which are antigenically similar to the vaccine components. LAIV was not efficacious against infection with circulating B strains, as most were mismatched to the lineage in the vaccine.

When differences in study year, and thus vaccine and circulating strains, are taken into account, our primary efficacy against vaccine-matched strains of 57·5% (95% CI 43·6–68·0) is very close to the 59·9% (31·1–77·4) efficacy against vaccine-matched strains reported for a single-dose Ann-Arbor-based LAIV given to children aged 2–3 years in southeast Asia.^16^ Among children aged 2 years in South Africa and South America, an Ann-Arbor-backbone LAIV had 71·5% (95% CI 52·9–83·4) and 81·8% (66·8–90·8) efficacy against laboratory-confirmed influenza illness after one or two doses, respectively.^17^ As the vaccine used in our study is licensed for single-dose administration,^10^ we did not include a two-dose group. The increased cost and logistical challenges of a second dose would need to be weighed against any incremental benefit in low-resource settings. If the Russian-backbone vaccine were integrated into vaccine programmes in most low-income and middle-income countries, use of one dose would be likely to have substantial logistical and cost advantages over a two-dose regimen. Future studies could explore the benefit of a second dose of vaccine in children receiving influenza vaccine for the first time.

The proportions of children in our study who received LAIV and had influenza illness were similar in Kamalapur (7·0%) and Matlab (6·1%), but the attack rate was higher in the Matlab placebo group than in the Kamalapur placebo group (21·9% *vs* 13·0%, table 2). Although the 95% CIs overlapped, vaccine efficacy for vaccine-matched strains was higher in Matlab than in Kamalapur in the per-protocol analysis set (72·0%, 95% CI 54·7–82·6 *vs* 46·2%, 23·0–62·4), possibly because of a lower force of infection in the rural setting than in the densely populated urban setting. Furthermore, the enrolled population in Kamalapur might have been less healthy than those in Matlab, since higher proportions of children had severe stunting and asthma or wheezing illness.

We chose our vaccination period to ensure children were vaccinated before the onset of peak influenza season, which is typically between April and September.^2^, ^4^ The efficacy we showed against the A(H1N1)pdm09 viruses is important because several observational studies of the Ann-Arbor-backbone LAIV in the USA in the 2013–14 and 2015–16 seasons and the Russian-backbone LAIV in Senegal showed no such efficacy.^18^, ^19^, ^20^ The same vaccine lot was used for this and the Senegal trial, and the storage conditions at both sites were well monitored and well maintained. Thus, a vaccine-specific cause for the difference in results seems unlikely. Influenza vaccine is not widely used in Bangladesh. Whether the lack of previous exposure to influenza vaccine, use of a different vaccine formulation than had been used previously, or both, affected influenza A(H1N1) efficacy in our Bangladesh population differently from that in the same period in the USA is unclear. It is likely, however, that excluding children who had previously been vaccinated ensured generalisability of our findings to the wider Bangladesh population. Unfortunately, the generalisability of our findings to other settings is uncertain.

The major limitation of our study is that it focused on clinical efficacy and did not include immunogenicity measurements. At the time of this study, the preferential use of LAIVs was being considered in several countries due to superior performance in young children in randomised trials over inactivated vaccines. Our study was done before the studies in Senegal and the USA showed lack of efficacy for A(H1N1)pdm09 strains. In retrospect, information on the immunogenicity and replicative ability of the vaccine used in this study might have informed our interpretation of the variation in results between settings that subsequently emerged. However, the overall usefulness of immunogenicity data in this context is uncertain because of the difficulty in identifying protective immunological correlates for LAIVs in previous studies.^21^

In a previous study of 300 children in Kamalapur, we prospectively assessed wheezing endpoints for the single-dose Russian-backbone LAIV.^8^ In this larger study, background wheezing was higher in urban Kamalapur (9·1%) than in rural Matlab (3·9%), but increases in or worsening of wheezing-related illness were not seen in either site. These results are reassuring, particularly in combination with a similar lack of wheezing signal in the Senegal study,^18^ and support careful assessment of the Russian-backbone LAIV in younger age groups. Previous data from Bangladesh show high attack rates and severe illness in young children^3^, ^4^, ^5^, ^7^ particularly those younger than 2 years.^4^, ^6^

This study corroborates data obtained over many years in urban and rural Bangladesh, which have shown sustained circulation of multiple influenza strains and associated high clinical attack rates.^2^, ^3^, ^4^, ^5^, ^6^, ^7^, ^12^, ^22^, ^23^, ^24^ Overall, 24·5% of children in our study had laboratory-confirmed influenza illness (any strain) during the course of this trial, including over a third (34·8%) of children at the rural Matlab site. Circulation of B viruses was common, which suggests that the use of a quadrivalent vaccine with efficacy against both B lineages might further increased the magnitude of the effect.

Our results support the use of a single-dose LAIV to prevent medically attended lower respiratory tract illness in young children in Bangladesh. Large multisite trials of vaccines in children done over multiple years could improve measurement of the effects of influenza vaccines, establish the burden of severe influenza disease in young children, and inform policy and financing decisions.^25^ Improved understanding of population-based differences in influenza vaccine performance is crucial to designing effective public health programmes in low-resource settings.

## Figures and Tables

**Table 1:** Baseline characteristics

		**Kamalapur**		**Matlab**		**All**		
		LAIV (n=800)	Placebo (n=400)	LAIV (n=374)	Placebo (n=187)	LAIV (n=1174)	Placebo (n=587)	Total (n=1761)
Mean (range) age (months)		42·7 (24–59)	42·3 (24–59)	42·2 (24–59)	41·8 (24–59)	42·5 (24–59)	42·1 (24–59)	42·4 (24–59)
Age group (years)								
	≥2 to <3	248 (31·0%)	138 (34·5%)	105 (28·1%)	53 (28·3%)	353 (30·1%)	191 (32·5%)	544 (30·9%)
	≥3 to <4	229 (28·6%)	105 (26·3%)	134 (35·8%)	69 (36·9%)	363 (30·9%)	174 (29·6%)	537 (30·5%)
	≥4 to <5	323 (40·4%)	157 (39·3%)	135 (36·1%)	65 (34·8%)	458 (39·0%)	222 (37·8%)	680 (38·6%)
Sex								
	Male	410 (51·3%)	191 (47·8%)	175 (4·8%)	90 (48·1%)	585 (49·8%)	281 (47·9%)	866 (49·2%)
	Female	390 (48·8%)	209 (52·3%)	199 (53·2%)	97 (51·9%)	589 (50·2%)	306 (52·1%)	895 (50·8%)
Underweight (weight for age malnutrition)*								
	None	203 (25·4%)	103 (25·8%)	106 (28·3%)	48 (25·7%)	309 (26·3%)	151 (25·7%)	460 (26·1%)
	Mild	324 (40·5%)	173 (43·3%)	162 (43·3%)	84 (44·9%)	486 (41·4%)	257 (43·8%)	743 (42·2%)
	Moderate	219 (27·4%)	105 (26·3%)	91 (24·3%)	43 (23·0%)	310 (26·4%)	148 (25·2%)	458 (26·0%)
	Severe	54 (6·8%)	19 (4·8%)	15 (4·0%)	12 (6·4%)	69 (5·9%)	31 (5·3%)	100 (5·7%)
Stunting (height for age malnutrition)*								
	None	152 (19·0%)	84 (21·0%)	111 (29·7%)	47 (25·1%)	263 (22·4%)	131 (22·3%)	394 (22·4%)
	Mild	275 (34·4%)	144 (36·0%)	140 (37·4%)	81 (43·3%)	415 (35·3%)	225 (38·3%)	640 (36·3%)
	Moderate	257 (32·1%)	127 (31·8%)	95 (25·4%)	50 (26·7%)	352 (30·0%)	177 (30·2%)	529 (30·0%)
	Severe	116 (14·5%)	45 (11·3%)	28 (7·5%)	9 (4·8%)	144 (12·3%)	54 (9·2%)	198 (11·2%)
Wasting (weight for height malnutrition)*								
	None	495 (61·9%)	249 (62·3%)	209 (55·9%)	100 (53·5%)	704 (60·0%)	349 (59·5%)	1053 (59·8%)
	Mild	243 (30·4%)	125 (31·3%)	120 (32·1%)	65 (34·8%)	363 (30·9%)	190 (32·4%)	553 (31·4%)
	Moderate	60 (7·5%)	20 (5·0%)	42 (11·2%)	21 (11·2%)	102 (8·7%)	41 (7·0%)	143 (8·1%)
	Severe	2 (0·3%)	6 (1·5%)	3 (0·8%)	1 (0·5%)	5 (0·4%)	7 (1·2%)	12 (0·7%)
Asthma or wheezing illness								
	Previous hospital admission	58 (7·3%)	20 (5·0%)	0	0	58 (4·9%)	20 (3·4%)	78 (4·4%)
	Previous treatment	242 (30·3%)	134 (33·5%)	0	0	242 (30·3%)	134 (33·5%)	376 (21·4%)

LAIV=live attenuated influenza vaccine.

* *Z* score, mild (−2 to <–1), moderate (−3 to <–2), or severe (<–3).

**Table 2:** Vaccine efficacy in the per-protocol population*

	**LAIV (n=1174)**		**Placebo (n=587)**		**Vaccine efficacy (95% CI)**
	Number of infections	Attack rate (%)	Number of infections	Attack rate (%)	
**Whole study population (n=1761)**					
All vaccine-matched strains	79	6·7%	93	15·8%	57·5% (43·6 to 68·0)
All strains	170	14·5%	144	24·5%	41·0% (28·0 to 51·6)
H1N1	21	1·8%	21	3·6%	50·0% (9·2 to 72·5)
H3N2	57	4·9%	72	12·3%	60·4% (44·8 to 71·6)
B/Yamagata (vaccine-matched)	2	0·2%	1	0·2%	0% (−1001·0 to 90·9)
B/Victoria (unmatched)	58	4·9%	31	5·3%	6·5% (−43·0 to 38·8)
**Kamalapur (n=1200)**†					
All vaccine-matched strains	56	7·0%	52	13·0%	46·2% (23·0 to 62·4)
With history of asthma or wheeze‡	22	8·9%	17	12·6%	29·0% (−29·0 to 60·9)
Without history of asthma or wheeze	34	6·1%	35	13·2%	53·4% (27·2 to 70·3)
All strains	103	12·9%	79	19·8%	34·8% (14·8 to 50·1)
**Matlab (n=561)**§					
All vaccine-matched strains	23	6·1%	41	21·9%	72·0% (54·7 to 82·6)
All strains	67	17·9%	65	34·8%	48·5% (30·9 to 61·5)

LAIV=live attenuated influenza vaccine.

* Includes laboratory-confirmed influenza infections occurring from 8 days onwards after receiving vaccine or placebo.

† n=800 in the LAIV group, n=400 in the placebo group.

‡ Vaccine efficacy analyses including history of asthma or wheezing could only be interpreted for the Kamalapur study site where history was identified at baseline. No participants at the Matlab site indicated a history of asthma or wheeze.

§ n=347 in the LAIV group, n=187 in the placebo group.

**Table 3:** Local and systemic reactions in the 7 days after vaccination and protocol-defined wheezing illness at any time

	**LAIV (n=1174)**			**Placebo (n=587)**			**Whole population (n=1761)**		
	Mild	Moderate	Severe	Mild	Moderate	Severe	Mild	Moderate	Severe
**Local and systemic reactions**									
Fever (≥38°C)	17 (1·4%)	20 (1·1%)	0	8 (1·4%)	8 (1·4%)	0	25 (1·4%)	28 (1·6%)	0
Nasal congestion	1 (0·1%)	0	0	1 (0·2%)	0	0	2 (0·1%)	0	0
Runny nose	68 (5·8%)	0	0	39 (6·6%)	0	0	107 (6·1%)	0	0
Cough	72 (6·1%)	1 (0·1%)	0	43 (7·3%)	0	0	115 (6·5%)	1 (0·1%)	0
Sore throat	4 (0·3%)	0	0	2 (0·3%)	0	0	6 (0·3%)	0	0
Ear pain	2 (0·2%)	0	0	3 (0·5%)	0	0	5 (0·3%)	0	0
Headache	0	0	0	1 (0·2%)	0	0	1 (0·1%)	0	0
Vomiting	4 (0·3%)	0	0	5 (0·9%)	1 (0·2%)	0	9 (0·5%)	1 (0·1%)	0
Chills	0	0	0	0	0	0	0	0	0
Irritability or decreased activity	1 (0·1%)	0	0	0	0	0	1 (0·1%)	0	0
Muscle/joint pain	3 (0·3%)	1 (0·1%)	0	0	0	0	3 (0·2%)	1 (0·1%)	0
Tachypnoea*	77 (6·6%)	9 (0·5%)	0	53 (9·0%)	1 (0·2%)	0	130 (7·4%)	10 (0·6%)	0
**Protocol-defined wheezing illness**									
Days 0–7	4 (0·3%)	0	0	1 (0·2%)	0	0	5 (0·3%)	0	0
Days 8–42	16 (1·4%)	0	0	9 (1·5%)	0	0	25 (1·4%)	0	0
Day 43 to 6 months	38 (3·2%)	1 (0·1%)	0	31 (5·3%)	1 (0·2%)	0	69 (3·9%)	2 (0·1%)	0
Day 0 to 6 months	53 (4·5%)	1 (0·1%)	0	37 (6·3%)	1 (0·2%)	0	90 (5·1%)	2 (0·1%)	0
Anytime†	78 (6·6%)	3 (0·3%)	0	46 (7·8%)	4 (0·7%)	0	124 (7·0%)	7 (0·4%)	0

LAIV=live attenuated influenza vaccine.

* Mild 31–40 breaths per min, moderate 41–50 breaths per min, and severe ≥51 breaths per min.

† Including events occurring >6 months after vaccination.
