# Supplementary material for: Efficacy of a Russian-backbone live attenuated influenza vaccine among young children in Bangladesh: a randomised, double-blind, placebo-controlled trial
Source: Lancet Glob Health. 2016 Oct 13;4(12):e946–54. doi: 10.1016/S2214-109X(16)30200-5 (PMC5118223; doi:10.1016/S2214-109X(16)30200-5)
Supplement: Supplementary appendix [file mmc1.pdf]

THE LANCET Global Health

Supplementary appendix

This appendix formed part of the original submission and has been peer reviewed. We post it as supplied by the authors.

Supplement to: Brooks WA, Zaman K, Lewis KDC, et al. Efficacy of a Russian-backbone live attenuated influenza vaccine among young children in Bangladesh: a randomised, double-blind, placebo-controlled trial. *Lancet Glob Health* 2016; published online Oct 13. http://dx.doi.org/10.1016/S2214-109X(16)30200-5.

Supplementary Appendix

Supplementary Table 1: Eligibility Criteria

**Inclusion Criteria**	**Exclusion Criteria**	**Temporary Contraindications***
Healthy male or female child at least 24 months of age and no older than 59 months of age at the time of study vaccination.	Serious, active, medical condition, including:<br>o chronic disease of any body system<br>o chronic infections such as tuberculosis<br>o genetic disorders, such as Down's syndrome or other cytogenetic disorder<br>o known or suspected disease of the immune system of any kind.	Acute illness accompanied by a body temperature of ≥38°C (axillary measurement).
A child whose parent or guardian's primary residence, at the time of study vaccinations, is within the Kamalapur surveillance site catchment area or Matlab service area and who intends to be present in the area for the duration of the trial.	History of documented hypersensitivity to eggs or other components of the vaccine (including gelatin, sorbitol, lactalbumin and chicken protein), or with life-threatening reactions to previous influenza vaccinations.	Any illness accompanied by active wheezing within 14 days of enrollment visit.
A child whose parent or legal guardian is willing to provide written informed consent prior to the participant's study vaccination.	Receipt of immunosuppressive agents, including systemic corticosteroids, during the month before planned study vaccination.	
	History of Guillain-Barré syndrome.	
	Receipt of aspirin therapy or aspirin-containing therapy within the two weeks before planned study vaccination.	
	History of any severe allergic reaction with generalized urticarial, angioedema, or anaphylaxis.	
	History of receiving influenza vaccine (LAIV or inactivated)	
	Has current or past participation (within 2 months of trial enrollment visit) in any clinical trial involving a drug or biologic with activity against respiratory disease.	
	Lives in household with somebody currently participating in a respiratory vaccination or antiviral study.	
	Has any condition determined by investigator as likely to interfere with evaluation of the vaccine or be a significant potential health risk to the child or make it unlikely that the child would complete the study.	

*Enrollment and administration of LAIV or placebo postponed until the child has recovered.

Supplementary Table 2: Vaccine Efficacy in the Per-Protocol Population*

	**LAIV (n=1174)**		**Placebo (n=587)**		**Vaccine efficacy (95% CI)**
	Number of infections	Attack rate (%)	Number of infections	Attack rate (%)	
**Whole study population**					
All vaccine-matched strains	87	7·4%	98	16·7%	55·6% (41·8 to 66·2)
All strains	178	15·2%	149	25·4%	40·3% (27·5 to 50·8)
H1N1	29	2·5%	27	4·6%	46·3% (10·1 to 67·9)
H3N2	57	4·9%	72	12·3%	60·4% (44·8 to 71·6)
B/Yamagata (vaccine-matched)	2	0·2%	1	0·2%	0% (–1001 to 90·9)
B/Victoria (unmatched)	58	4·9%	31	5·3%	6·5% (–43·0 to 38·8)
**Kamalapur (n=1200)†**					
All vaccine-matched strains	60	7·5%	56	14·0%	46·4% (24·4 to 62·0)
All strains	107	13·4%	83	20·8%	35·5% (16·4 to 50·3)
**Matlab (n=561)‡**					
All vaccine-matched strains	23	7·2%	42	22·5%	67·9% (49·6 to 79·5)
All strains	71	19·0%	66	35·3%	46·2% (28·4 to 59·6)

LAIV=live attenuated influenza vaccine. *Includes laboratory-confirmed influenza infections occurring at any time after receiving vaccine or placebo. †n=800 in the LAIV group, n=400 in the placebo group. ‡n=347 in the LAIV group, n=187 in the placebo group.

Supplementary Table 3: Reactions

Local Reactions	Systemic Reactions	Severity
Runny nose<br>Sore throat<br>Stuffy nose<br>Ear pain<br>Cough	Chills<br>Headache<br>Vomiting<br>Fever (subjective)<br>Irritability / Decreased activity<br>Muscle/joint pain	Mild: events require minimal or no treatment and do not interfere with child's functioning
		Moderate: events result in low level of concern with therapeutic measures. May cause some interference with normal functioning
		Severe: events interrupt child's functioning and may require systemic drug therapy or other treatment. Severe events are usually incapacitating
		Life threatening: any adverse experience that places the child, in the view of the investigator, at immediate risk of death
	Fever (measured)	Mild: 38·0°C – 38·4°C axillary
		Moderate: 38·5°C – 39·9°C axillary
		Severe: ≥40°C axillary
	Tachypnea	Mild: respiratory rate 31 – 40 breaths/min
		Moderate: respiratory rate 41 – 50 breaths/min
		Severe: respiratory rate ≥51 breaths/min
